# Nonradiating anapole modes in dielectric nanoparticles

**DOI:** 10.1038/ncomms9069

**Published:** 2015-08-27

**Authors:** Andrey E. Miroshnichenko, Andrey B. Evlyukhin, Ye Feng Yu, Reuben M. Bakker, Arkadi Chipouline, Arseniy I. Kuznetsov, Boris Luk'yanchuk, Boris N. Chichkov, Yuri S. Kivshar

**Affiliations:** 1 Nonlinear Physics Centre, The Australian National University, Acton, Australian Capital Territory 2601, Australia; 2 Laser Zentrum Hannover e.V., Hollerithallee 8, D-30419 Hannover, Germany; 3 Institute of Laser and Information Technologies RAS, 142190 Moscow, Troitsk, Russia; 4 Data Storage Institute, A*STAR, 5 Engineering Drive 1, 117608, Singapore; 5 Technische Universität Darmstadt, Institut für Mikrowellentechnik und Photonik, Merckstraße 25, 64283 Darmstadt; 6 Saint-Petersburg Polytechnic University, Polytechnicheskaya 29, 195251 St Petersburg, Russia

## Abstract

Nonradiating current configurations attract attention of physicists for many years as possible models of stable atoms. One intriguing example of such a nonradiating source is known as ‘anapole'. An anapole mode can be viewed as a composition of electric and toroidal dipole moments, resulting in destructive interference of the radiation fields due to similarity of their far-field scattering patterns. Here we demonstrate experimentally that dielectric nanoparticles can exhibit a radiationless anapole mode in visible. We achieve the spectral overlap of the toroidal and electric dipole modes through a geometry tuning, and observe a highly pronounced dip in the far-field scattering accompanied by the specific near-field distribution associated with the anapole mode. The anapole physics provides a unique playground for the study of electromagnetic properties of nontrivial excitations of complex fields, reciprocity violation and Aharonov–Bohm like phenomena at optical frequencies.

## Abstract (web summary)

The anapole is an intriguing example of a nonradiating source useful in the study of electromagnetic properties in complex phenomena. Here, Miroshnichenko *et al.* demonstrate that a single dielectric nanoparticle can exhibit a radiationless anapole mode at visible wavelengths.

## Graphical abstract

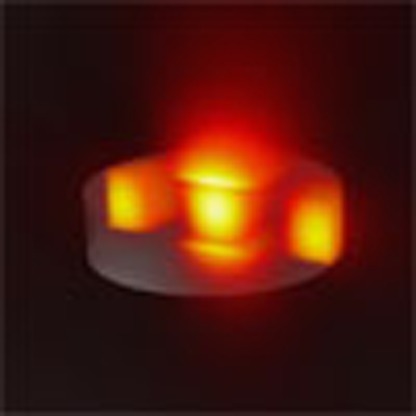

The possible existence of non-radiating sources has puzzled physicists since the early days of electromagnetic theory, particularly in connection with models of stable atoms and electrons configurations^1^,^2^,^3^. The term ‘anapole' (that means ‘without poles' in Greek) was introduced in the physics of elementary particles by Yakov Zel'dovich^4^. Recently, an anapole was suggested as a classical model of elementary particles describing dark matter in the Universe^5^. The electrodynamics analogue of the anapole is a composition of electric and toroid dipole moments, without far-field radiation due to complete destructive interference of their similar radiation patterns^6^. The toroidal modes and an analogue dynamic anapoles have been demonstrated in the microwave part of the spectrum^7^, toroidal resonances were also seen at optical frequencies^8^. The classical analogue of a stationary anapole is the well-known toroid with a constant poloidal surface current^4^. This current distribution is also associated with a toroidal dipole moment pointing outward along the torus symmetry axis (see Fig. 1). The static magnetic field produced by a toroid is entirely concentrated within a coil in the form of a circulating magnetic current (see Fig. 1). It generates no field outside, but may possess a non-zero potential, which might lead to violation of the reciprocity theorem and Aharonov–Bohm like phenomena^9^,^10^.

In general, the existence of toroidal multipoles is required by the symmetry of the order parameters with respect to the inversions of space and time: in addition to the well-known electric polarization (described by an r-odd and t-even polar vector) and magnetization (r-even and t-odd axial vector), there should be the order parameters described by an r- and t-even polar vector and an r- and t-odd axial vector^11^. The latter two correspond to the toroidal ordering and have been observed in natural crystals (see, for example, ref. 12, for a recent review) as well as in artificial metamaterials^13^. A radiationless nuclear anapole moment has been observed in atomic Cesium^14^ through atomic parity-violating effects, which were first suggested in ref. 15. In the Standard Electroweak theory, nuclear anapole moments arise due to parity-violating forces inside the nucleus. However, the interactions of nucleons with a static ‘cosmic' field and an axion dark matter field can also give rise to static and oscillating nuclear anapole moments, respectively, (see for example, refs 16, 17).

In the dynamic case, an oscillating toroidal dipole moment produces non-zero electromagnetic radiation with a pattern fully repeating that of an electric dipole moment but being scaled by a factor *ω*^2^, where *ω* is the angular frequency of light (see Fig. 1). For the oscillating surface current, its radiationless properties can be realized by exciting a second electric dipole oscillating out-of-phase with the toroid, resulting in complete destructive interference of their radiation due to equivalence of their scattering patterns. Such a radiationless nontrivial oscillating current configuration was (in analogy with non-oscillating poloidal current) also named ‘anapole'^6^. However, this type of the radiation compensation, in general, is not complete for an external excitation: the compensated toroidal dipole moment is a part of the third-order source current multipole expansion. Other terms of the same order (magnetic quadrupole and electric octupole) can remain radiative. Nontrivial nonradiative current configurations have attracted continuous interest since the early days of electrodynamics; from the fundamental physics to applications of nonscattering objects^18^,^19^. Since ideal nonscattering objects do not exist^20^, by ‘radiationless' we refer to a compensation or absence due to structural peculiarities of the leading multipole order; below we term the respective combination as the ‘anapole mode'.

The concept of toroidal modes have attracted considerable attention in the field of metamaterials as a possible realization of radiationless objects^7^,^21^,^22^,^23^,^24^,^25^. The toroidal moment itself and its associated effects (including toroidal metamaterials^13^,^25^) have been recently studied theoretically^8^,^22^,^23^,^26^,^27^,^28^,^29^. Several experimental verifications in microwave^7^,^13^,^21^ and optical^30^ domains for toroidal moments have confirmed the proposed theory. Moreover, the mutual compensation of the toroidal and dipole moments has been experimentally observed in GHz frequency region with a specially designed structure^7^.

Here we demonstrate experimentally, for the first time to our knowledge, the existence of an anapole mode in optics in the simple structure of a silicon nanodisk. We observe a strong suppression of the far-field scattering along with nontrivial evolution of the electromagnetic field inside a nanodisk close to the wavelength of the anapole mode excitation. Very recently, excitation of a toroidal moment at THz frequencies has been theoretically predicted for quadrumers of dielectric cylinders made of LiTaO3 (ref. 8).

## Results

To analyze the electromagnetic properties of the silicon nanoparticle theoretically and to demonstrate the anapole excitation in the system, we employ multipole expansions in the two representations: for fields outside the nanoparticle in the spherical multipoles^31^ and in the Cartesian for currents inside the nanoparticle; each series can be unambiguously expressed one through the other^30^. For the sake of generality, we assume a nontrivial current distribution **J**(**r**, *t*) producing an electromagnetic field **E**(**r**, *t*).

We employ the field multipole expansion, which, due to orthogonally of vector spherical harmonics, allows us to unambiguously study the radiation properties by representing the total scattering cross-section as a sum of intensities of spherical electric *a*_E_(*l*, *m*) and magnetic *a*_M_(*l*, *m*) scattering coefficients^32^:


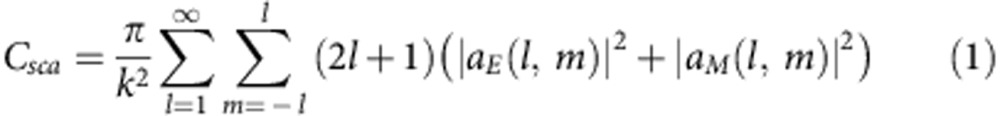


where the scattering coefficients


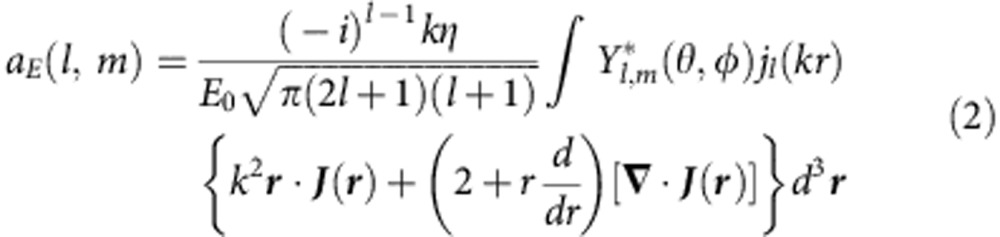



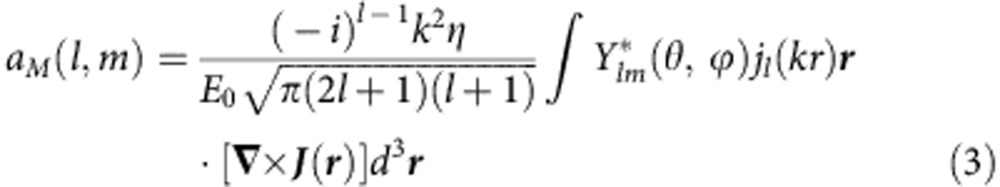


can be calculated directly through the induced currents inside the structure^31^. The coefficients *a*_E_(*l*, *m*) and *a*_M_(*l*, *m*) are called field spherical multipole coefficients^32^ (see also Supplementary Methods).

At the same time, the source current distribution can be expanded over current Cartesian multipole coefficients^26^,^33^,^34^:


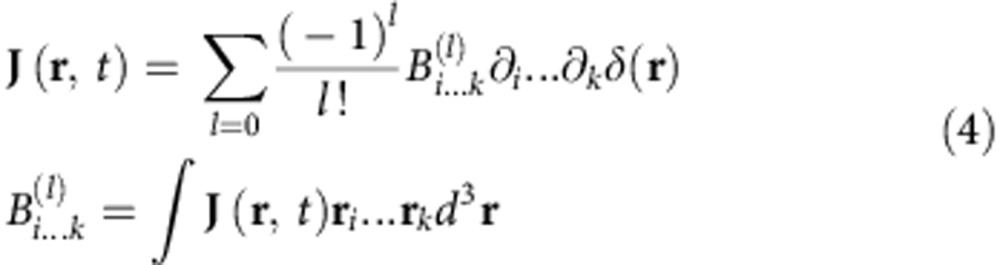


Here 
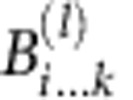
is a tensor of rank *l*. We would like to note that the structure and interpretation of these coefficients requires a special care. In particular, from these tensors, various current Cartesian multipoles can be obtained. For example, 
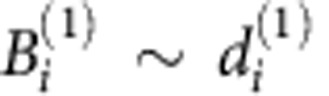
determines the electric dipole moment 
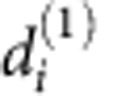
, 
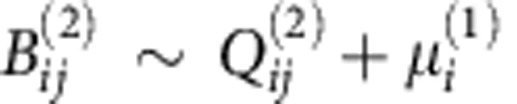
 consists of electric quadrupole 
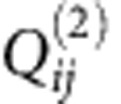
(symmetric) and magnetic dipole 
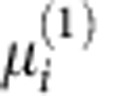
 (anti-symmetric) moments, and 
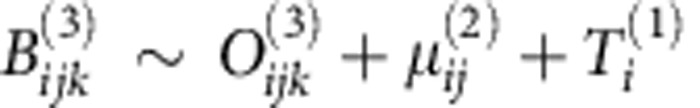
 gives rise to electric octupole 
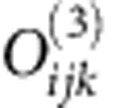
, magnetic quadrupole 
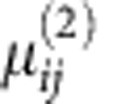
, and toroidal dipole moments 
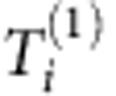
. Note that the toroidal dipole moment of the current appears in the third-order expansion coefficients^33^, in contrast to the field expansion, and is associated with a particular current configurations. Field expansion and respective field spherical multipole coefficients *a*_E_(*l*, *m*) and *a*_M_(*l*, *m*) are not associated with a particular current, but rather with the scattered field structure. For example, in the far field, the dipole and toroidal dipole radiation patterns are indistinguishable and cannot be recognized in the frame of the field spherical multipole expansion. From the other perspective, current Cartesian multipole expansion allows us to unambiguously separate the dipole and toroidal dipole contributions, and, hence, provides with a tool necessary for the required structure design. The current expansion can also be performed over any full orthogonal basis, for example over vector spherical harmonics^9^, where the contribution of the toroidal moments and corresponding current configurations can be uniquely identified.

We start our analysis from a simple observation of some peculiar properties of light scattering by spherical dielectric particles. In the Mie theory, the transverse boundary conditions can be written independently for each multipole order as ref. 35


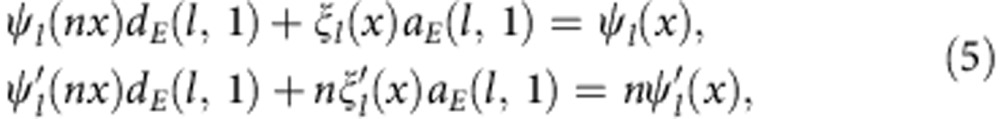


where *x*=2*πR*/*λ* is the size parameter, *n* is the relative refractive index of the particle, 
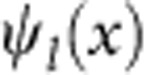
 and 
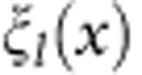
 are Riccati–Bessel functions and *d*_E_(*l*, 1) are the internal coefficients of electric multipole excitation inside the spherical particle^31^. In the limit of a transparent particle (*n*→1), all the scattering coefficients vanish *a*_E_(*l*, 1)=0 and the amplitude of the internal coefficients tend to unity, |*d*_E_(*l*, 1)|=1. This is a trivial case of a nonscattering particle without induced polarization inside. In the nontrivial case with *n*>1, in general, there are conditions when *a*_E_(*l*, 1)=0 and |*d*_E_(*l*, 1)|>1, corresponding to the situations with non-zero induced polarization inside the particle and, at the same time, zero scattering of a particular multipolar order. This condition exhibits the existence of non-radiative sources in the simplest spherical geometry. Since, for each multipole there exist an infinite number of such conditions, they provide a recipe to create a variety of nonradiative current configurations. In Fig. 2a we show the dependence of electric dipole scattering |*a*_E_(1, 1)| and internal |*d*_E_(1, 1)| coefficients of a dielectric spherical particle as a function of diameter for refractive index *n*=4 and wavelength 550 nm, which demonstrates the situation of a vanishing scattering coefficient *a*_E_(1, 1)=0 associated with the non-zero induced field. At the same time, in Fig. 2b we present the partial scattering contribution of the electric dipole *C*_*sca*_∝|*a*_*E*_(1, 1)|^2^ together with the corresponding electric energy 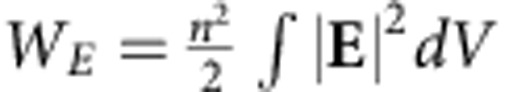 integrated over the spherical volume normalized to the trivial case of a transparent particle *W*_*in*_=2*πR*^3^|*E*_0_|^2^/3. It again demonstrates that at the condition of zero scattering the energy density is non-zero and larger than in trivial case—it means that the energy is concentrated inside the particle.

To analyse the excitation of the anapole mode in more details, we consider the properties of a single-spherical electric dipole. In this case, the response of the electric dipole mode can be also described in terms of effective dipole moments. The contribution to the far-field scattering with the electric dipole symmetry can be written as


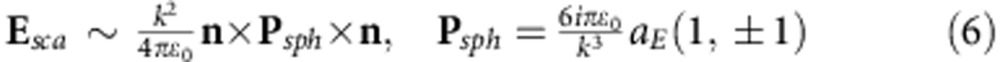


where **n** is the normalized vector of observation direction and the effective electric dipole moment is defined through the corresponding Mie scattering coefficient. This is a description of the radiation properties of an electric dipole by using a field spherical multipole coefficient.

However, electric dipole moments inside the particle can be obtained via integrating the induced current over the whole volume


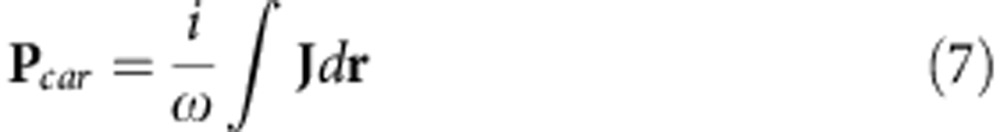


with


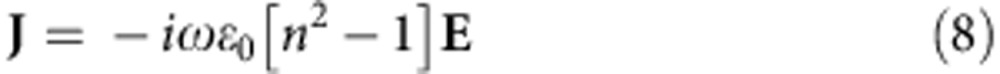


This is a description of the electric dipole moments inside the particle by using the current Cartesian multipoles. In Fig. 2c we plot the dependence of both electric dipole moments for a dielectric sphere versus diameter, where only the electric field of the spherical electric dipole inside the particle was taken into account. This figure demonstrates that for small particle sizes both approaches produce similar results. For larger particles, the two descriptions deviate from each other quite strongly. In particular, there is a situation for the diameter ∼204 nm, when the electric dipole scattering vanishes, **P**_*sph*_=0, while the induced electric dipole inside the particle is non-zero, |**P**_*car*_|≠0. In this case we have an apparent contradiction that there is a source of the electric dipole response, but no contribution to the far-field. Similarly, an opposite situation exists for the diameter around 196 nm, when the induced electric dipole moments inside the particle vanishes, **P**_*car*_=0, while there is a non-zero contribution to the far-field |**P**_*sph*_|≠0. In this case, we have a finite contribution to the far-field in the absence of a source! To resolve such contradictions, we note that for larger diameters, the electric field distribution inside the particle becomes highly inhomogeneous; simple averaging of the induced current (proportional to the electric field) is not enough to properly describe the contribution to the far-field. As was mentioned above, in general, the scattering coefficients can be expressed through Cartesian multipole coefficients 
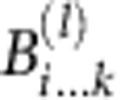
. The relation between current Cartesian and field spherical multipoles for the electric dipole coefficient *a*_*E*_ can be written as ref. 32


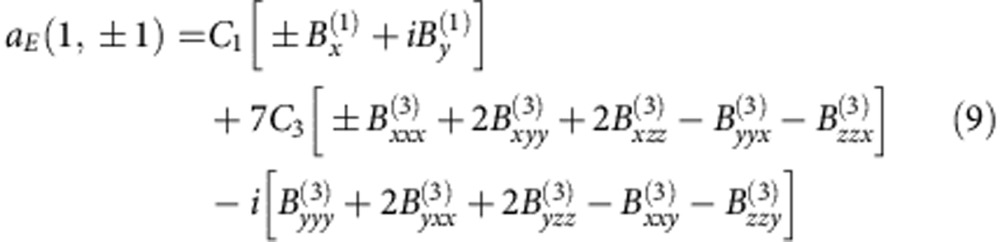


where *C*_1_=−*ik*^3^/(6*π*ɛ_0_*E*_0_) and *C*_3_=−*ik*^5^/(210*π*ɛ_0_*E*_0_) (ref. 30). We notice, that among these higher order current Cartesian coefficients there are toroidal dipole moments 
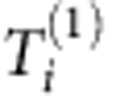
with a radiation pattern similar to the electric dipole (see, for example, Supplementary Information in ref. 33). It implies that it now should be taken into account to correctly describe the scattering properties. The toroidal dipole moment can be calculated via induced current as


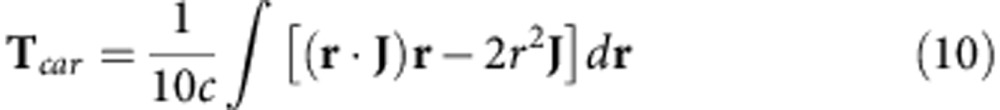


and the total contribution to the far-field scattering can now be written as (see Supplementary Methods)


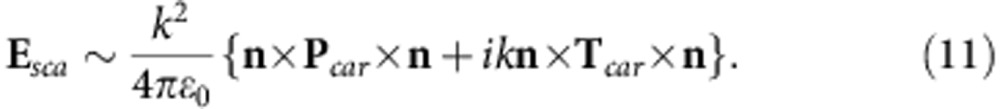


Thus, the far-field radiation vanishes, **E**_sca_=0, when the contributions of the current Cartesian electric and toroidal dipoles to the scattered field are out-of-phase


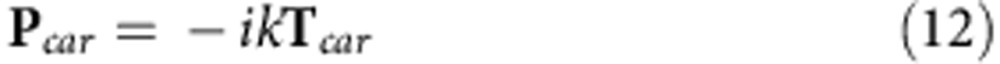


and, thus, interfere destructively with each other. This is the exact condition for excitation of the radiationless anapole mode.

In Fig. 2c we also show the dependence of the toroidal dipole contribution to the far-field, which becomes essential for larger sized particles. In particular, when the induced electric dipole moment vanishes, P_*car*_=0, only the toroidal dipole contributes to the far-field scattering. Alternatively, the scattering cancellation is possible, **P**_*sph*_=0, when non-zero electric and toroidal dipoles contributions compensate each other in the far-field. Thus, the partial scattering can vanish meaning that the electric dipole scattering coefficient becomes zero, *a*_*E*_(1,±1)≈0, which can be achieved when the first-order (electric dipole) coefficients 
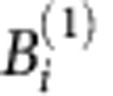
 have to be compensated by the toroidal part of the third-order coefficients 
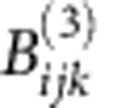
. This simple consideration creates the basis for understanding of physics of the anapole mode, namely, mutual compensation of lower 
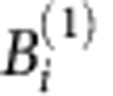
and part of the higher 
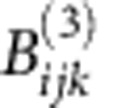
order components with a nontrivial current distribution inside the particle.

All of this raises an important question: is it possible to experimentally observe the anapole mode? By using external sources it is not possible to achieve total zero scattering due to Lorentz reciprocity. But, can we get around this condition with realistic structures? The spherical geometry under planewave illumination is not suitable, since in the vicinity of the anapole excitation other multipoles will produce strong contributions to the total scattering due to superposition. For successful experimental observation of the anapole we need to find a structure, which exhibits leading contribution of solely the electric dipole with all other modes being strongly suppressed. One of the possibilities to achieve this is to use a disk geometry with a particular aspect ratio. Specifically, we found that a silicon nanodisk of height 50 nm and diameter ranging from 200 to 400 nm supports the anapole mode, which can be experimentally excited and observed (see Fig. 3). For this geometry, calculations are made in CST Microwave Studio show a strong dip in the far-field scattering spectrum (see Fig. 3b), accompanied by a near-field enhancement inside and around the disk (see Fig. 3a), indicating the presence of the combination of electric and toroidal dipoles which provide the anapole mode configuration. Opposite circular displacement currents in the left and right hand sides of the disk (see Fig. 3c) generate a circular magnetic moment distribution that is perpendicular to the disk surface (see Fig. 3d). This provides a strong toroidal moment oriented parallel to the disk surface.

**Figure 1:**
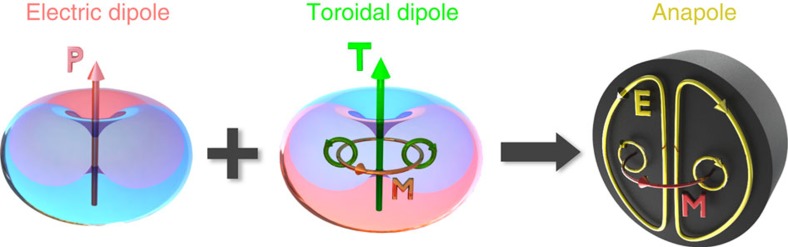
Illustration of an anapole excitation. The toroidal dipole moment is associated with the circulating magnetic field **M** accompanied by electric poloidal current distribution. Since the symmetry of the radiation patterns of the electric **P** and toroidal **T** dipole modes are similar, they can destructively interfere leading to total scattering cancelation in the far-field with non-zero near-field excitation.

**Figure 2:**
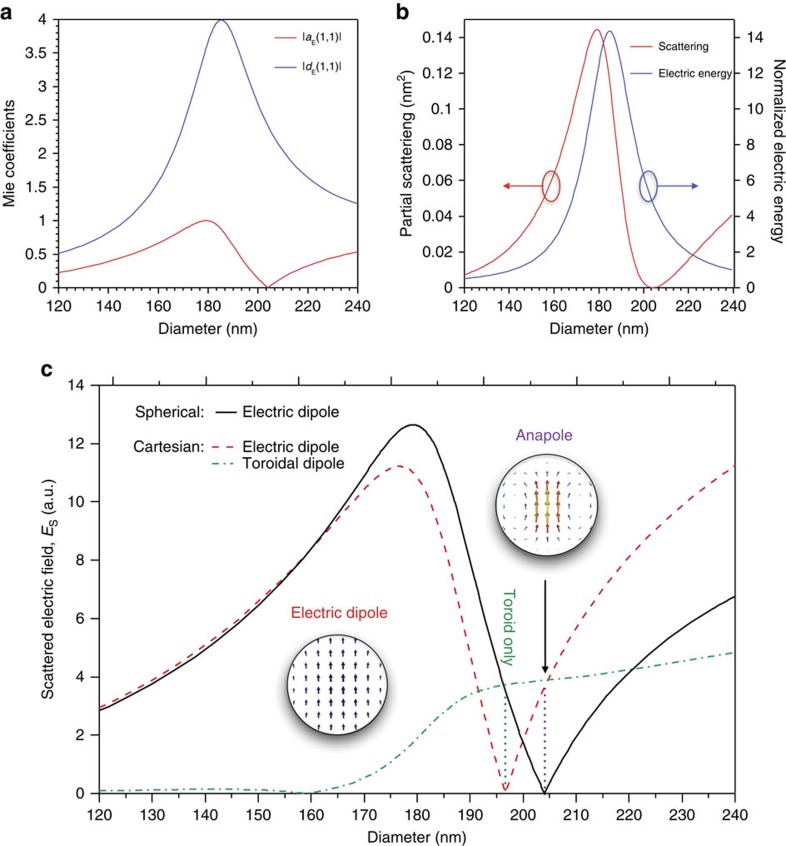
Decomposition of the scattering in Spherical and Cartesian multipoles. We consider scattering by a dielectric spherical particle inside as a function of diameter for refractive index *n*=4 and wavelength 550 nm: (**a**) Scattering |*a*_*E*_(1, 1)| and internal |*d*_*E*_(1, 1) Mie coefficients; (**b**) partial scattering cross-section and energy density of the electric dipole; (**c**) calculated spherical electric dipole |**P**_*sph*_|(black), Cartesian electric |**P**_*car*_| (red) and toroidal |**T**_*car*_| (green) dipole moments contributions to the partial scattering. These figures demonstrate that for small particles both contributions of the spherical and Cartesian electric dipoles are identical and the toroidal moment is negligible. For larger sizes, the contribution of the toroidal dipole moments to the total scattered field has to be taken into account. The anapole excitation is associated with the vanishing of the spherical electric dipole **P**_*sph*_=0, when the Cartesian electric and toroidal dipoles cancel each other.

**Figure 3:**
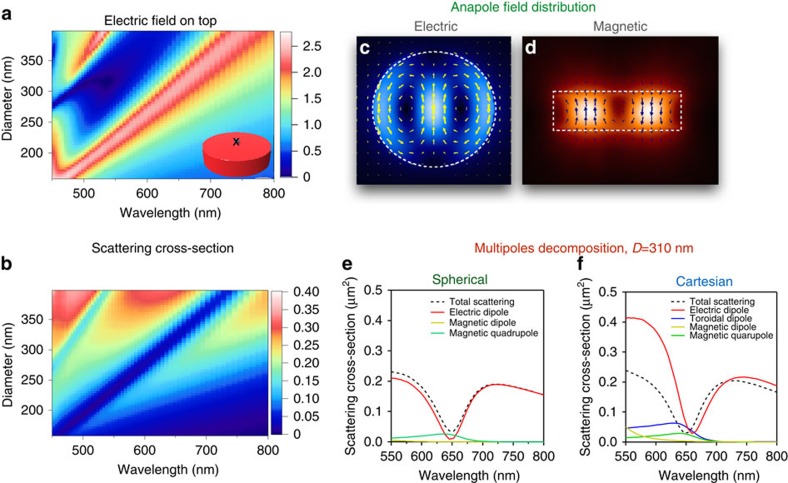
Numerical results for a single silicon nanodisk under normal incidence. (**a**) Electric field on top of the nanodisk of thickness h=50 nm at the surface centre for various diameter. (**b**) Total scattering cross-section spectra of the silicon nanodisks with similar parameters. The scattering suppression is accompanied by the electric field enhancement in the centre of the nanodisk. (**c**,**d**) Electric and magnetic scattered near-field distribution at the anapole wavelength corresponding to the mimimum of the far-field scattering. (**e**,**f**) Spherical and Cartesian multipole decompositions of the scattering spectra for diameter 310 nm. In field spherical harmonics only the electric dipole is dominant, which becomes suppressed at 650 nm wavelength. On contrary, current Cartesian multipoles exhibit two leading contributions from electric **P**_*car*_ and torodial **T**_*car*_ dipoles, which are out of phase and, thus, compensate each other in the far-field.

Excitation of this toroidal dipole and its destructive interference with the electric dipole is confirmed through current Cartesian and field spherical multipole decompositions (see the 310-nm diameter disk in Fig. 3e,f). One advantage of such disks compared with other geometries, for example, spheres, is that the other multipoles apart from the electric and toroidal dipoles are suppressed (see also Supplementary Fig. 1 for a silicon disk with diameter of 200 nm). We clearly observe a strong suppression of the total scattering due to the far-field cancelation of electric and toroidal dipole radiations (see Fig. 3f), which is exactly the condition for anapole mode excitation. Since we are exciting the silicon disk with an incident plane wave, the total scattering cannot completely vanish due to reciprocity. Instead, magnetic quadrupole radiation is dominant in the far-field (see Fig. 3e). At the same time, the near-field distribution is quite complex and does not correspond to a magnetic quadrupole only. The multipole expansion of current distribution inside the silicon nanodisks (see Supplementary Methods) indicates dominant contributions of electric and toroidal dipole moments (see Fig. 3f). The presence of magnetic quadrupole in disk geometry can be also understood from the structure of the toroidal dipole moment, schematically presented in Fig. 1. In particular, for an ideal spherical geometry, a toroidal dipole is characterized by the presence of the circulating magnetic flux along the closed loop. By transforming a sphere to a disk, this loop discontinues and leads to the formation of two anti-parallel magnetic moments, resulting in magnetic quadrupole radiation, which does not affect the condition of the anapole mode excitation. Our direct numerical results indicate that the anapole excitation in silicon disks is robust against incident angle and polarization (see also Supplementary Fig. 2).

**Figure 4:**
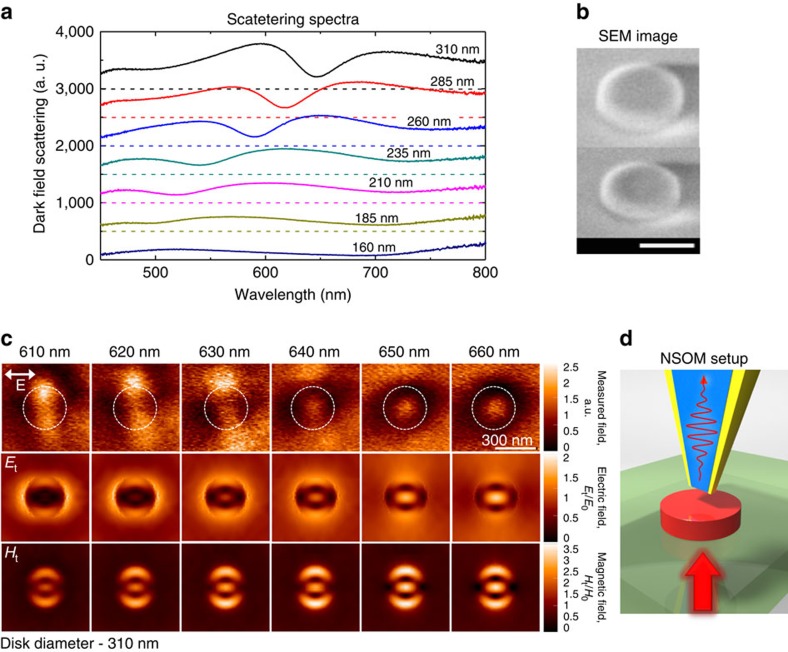
Experimental demonstration of the anapole mode in a silicon nanodisk. (**a**) Experimental dark field scattering spectra of silicon nanodisks with a height of 50 nm and a diameter ranging from 160 to 310 nm. The baseline for each spectrum has an offset step of 500 A.U. (**b**) SEM images of the two largest disks (for viewing convenience the view is tilted at 52°). The scale bar, 200 nm. (**c**) Near-field enhancement around the silicon nanodisk with diameter of 310 nm; the top row shows experimental NSOM measurements while the middle and bottom rows show calculated transversal electric and magnetic near-field, respectively, on top of the disk, 10 nm above the disk surface. White dashed lines in the experimental images indicate the disk position. Polarization of the excitation light is shown in the figure. (**d**) Schematic representation of NSOM measurement with the incident light coming through the substrate and collecting on the top with the metal-coated tip.

To confirm these theoretical predictions, we performed a series of experiments to observe the anapole excitation. Silicon nanodisks with a height of 50 nm and diameters ranging from 160 to 310 nm were fabricated on a quartz substrate using standard nanofabrication techniques (see Methods). Far-field scattering spectra (Fig. 4a) were measured using single nanoparticle dark-field spectroscopy (see Methods). For disks with a diameter >200 nm, a scattering dip appears around 550 nm; as the diameter increases, the dip redshifts and becomes more pronounced. The spectral position of this far-field scattering minimum is in good agreement with the theoretically predicted anapole excitation (see Fig. 3b for comparison). To show that the spectral dip in far-field scattering corresponds to the dark anapole mode excitation, the near-field distribution around the disks is mapped at multiple wavelengths using a near-field scanning optical microscope (NSOM) and a supercontinuum light source (see Methods). The observed deviation from the theoretically predicted suppression is primarily attributed to the non-perfect disk shape and inevitable roughness. Representative experimental near-field maps for the 310-nm diameter disk at wavelengths around the anapole excitation are provided in Fig. 4b, compared directly with simulated near-field maps of transverse electric and magnetic field at a height of 10 nm above the nanodisk. The numerical simulations were performed by using CST Microwave Studio for a bar silicon nanodisk and the near-field was collected above the nanoparticle for linear polarized planewave excitation coming from the bottom. In the simulations we did not take the presence of the probe into account. It is known, that the signal collected by an aperture-type near-field probe is not directly proportional to any of the near-field components separately but is a result of a convolution integral of both electric and magnetic near-fields (see for example, ref. 36). Thus, it should reflect near-field maxima of both electric and magnetic field components. For this reason in Fig. 4 we compare simulated electric and magnetic near-fields with the experimental NSOM data. The experimental near-field maps show the evolution of the spectral response throughout the visible. As the wavelength approaches 620 nm, we begin to see the splitting of the central hotspot into two separate spots. Close to the anapole mode wavelength, at 640 nm a new hotspot appears in the middle of the disk and its intensity increases with wavelength. Experimental results clearly show the appearance of a near-field maximum at the anapole wavelength in the middle of the disk, which corresponds to appearance of the maximum of electric near-field in simulations. The experimental results also show particular symmetry of the near-field excitation with main lobes aligned perpendicular to polarization direction. This is also in a good correlation with both electric and magnetic near-field components in the simulations. Similar near-field behaviour with an anapole excitation at 620 nm is observed for a disk with diameter of 285 nm (see Supplementary Fig. 3).

In summary, the anapole mode observed in the silicon nanodisk originates from the interference of two different dipole moments. Such radiationless excitation can make the nanodisk almost invisible in the far-field at the anapole's excitation wavelength. The anapole mode offers a new way to achieve an invisibility condition for lossless dielectric nanostructures based on the cancellation of radiation scattering (originally proposed by Kerker in (ref. 37) for metallic core-shell nanoparticles). Moreover, the relation between the electric and toroidal dipoles, including their mutual cancelation in the far-field can be directly extended to magnetic moments, when the magnetic dipole radiation vanishes. Recent observations of a strong magnetic dipole response from silicon nanoparticles^38^,^39^,^40^ suggests that it could be an ideal platform for the demonstration of such magnetic type anapoles.

We also mention that the anapole mode is not only limited to disk geometries but can also be observed for spheres^38^ or other dielectric nanostructures where the electric dipole contribution vanishes due to excitation of other modes with electric dipole symmetry in the far-field. For other geometries, however, this effect can be hidden by contributions of higher-order multipole modes (quadrupoles, octupoles and so on). Similar effects can also be expected in metallic systems supporting ‘higher-order dipole' modes.

## Methods

### Fabrication

Silicon nanodisks with various diameters were fabricated on quartz substrates by standard nanofabrication techniques. A 50-nm silicon film was grown on a quartz substrate using chemical vapor deposition (ICP-CVD, Oxford Instruments). A thin (<60 nm) layer of negative resist (HSQ) was coated on the sample. Lithography was performed with an electron beam lithography machine (Elionix 100 KV). After electron beam exposure and development, reactive ion etching (ICP, Oxford Instruments) was used to transfer the pattern into the silicon film. The result is silicon disks with a thin (<10 nm) cap of residual resist on top of a quartz substrate.

### Far-field spectroscopy

Spectral analysis was performed using a single-nanoparticle spectroscopy setup with a dark-field geometry (see ref. 40 for details). The sample was irradiated by a halogen lamp source at an angle of 58.5°. According to our simulations the spectral position of the anapole excitation practically does not change for variations of the incidence angle in the range from 0° to 60° (see Supplementary Fig. S3). Scattered light is collected from above into a solid angle corresponding to the microscope objective lens with 0.55 numerical aperature. The collected scattering spectra were normalized to the halogen lamp spectrum measured in a bright-field reflection geometry.

### Near-field

The sample was characterized in the near-field using a NSOM (Multiprobe SPM/NSOM Cryoview, Nanonics Imaging). The sample was illuminated from the far-field and the near-field was collected through a subwavelength aperture (50-nm aperture of a tapered fibre, coated with Chrome and Gold) in a transmission configuration. The light source used is a supercontinuum source (SuperK Power, NKT Photonics). Specific wavelengths were selected using a variable bandpass filter (SuperK Varia, NKT Photonics). Photons were counted with Avalanche Photo Diodes (Excelitas Technologies). Scans on individual particles were performed with various wavelengths over a 2 × 2 μm area with a pixel size of 8 nm. The near-field maps are normalized, taking the background as unity.

## Additional information

**How to cite this article:** Miroshnichenko, A. E. *et al.* Nonradiating anapole modes in dielectric nanoparticles. *Nat. Commun.* 6:8069 doi: 10.1038/ncomms9069 (2015).

## Supplementary Material

Supplementary Information Supplementary Figures 1-3, Supplementary Methods, Supplementary References.
