# Supplementary material for: Nonradiating anapole modes in dielectric nanoparticles
Source: Nat Commun. 2015 Aug 27;6:8069. doi: 10.1038/ncomms9069 (PMC4560796; doi:10.1038/ncomms9069)
Supplement: Supplementary Information — Supplementary Figures 1-3, Supplementary Methods, Supplementary References. [file ncomms9069-s1.pdf]

Supplementary Figures

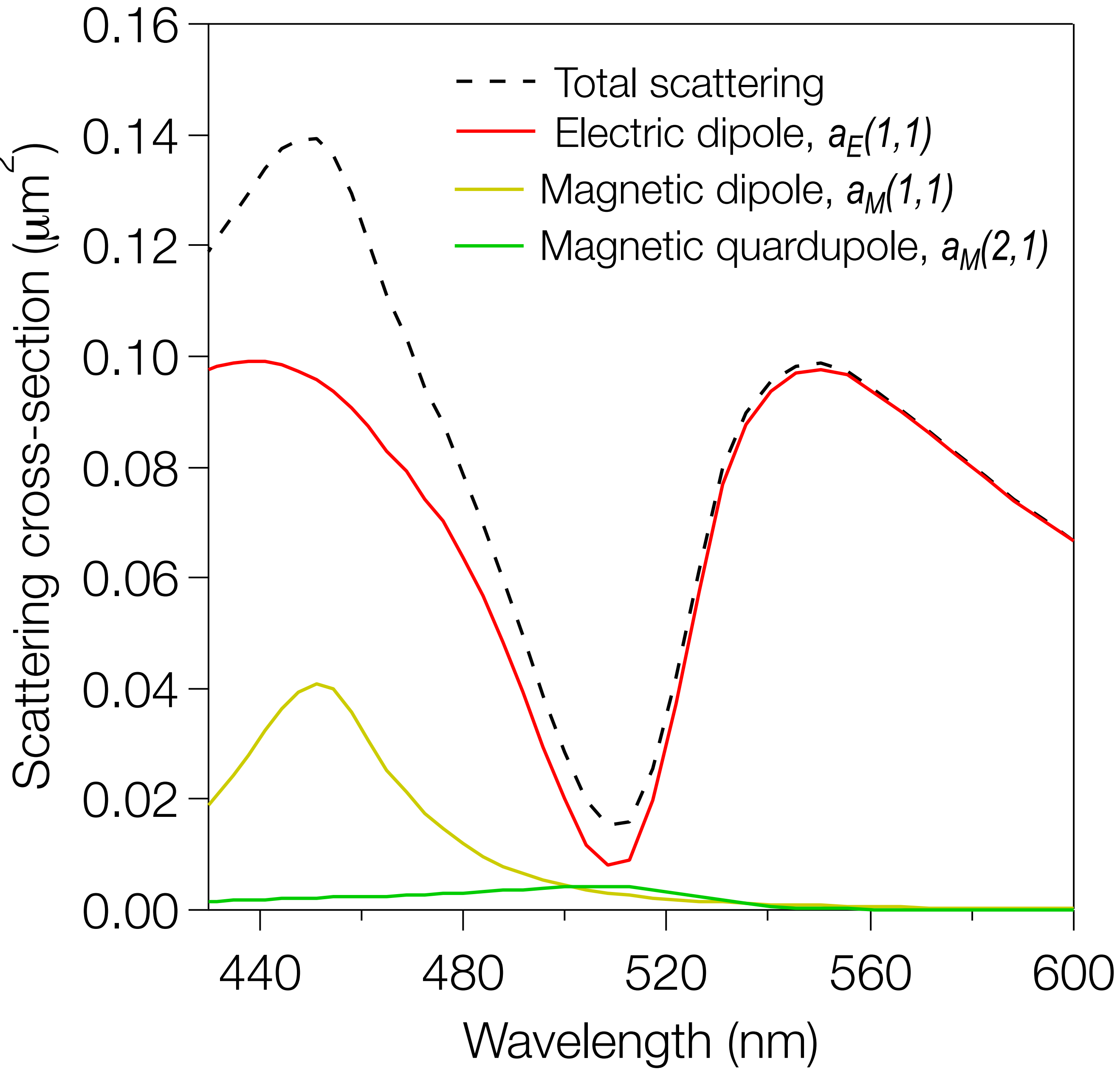

Supplementary Figure 1: **Spherical multipole decomposition of the scattering spectra of a silicon nanodisk**. In this case, at the anapole excitation the dominant contribution is the electric dipole only, while all other modes are significantly suppressed. The height of the silicon nanodisk is 50 nm and diameter 200 nm.

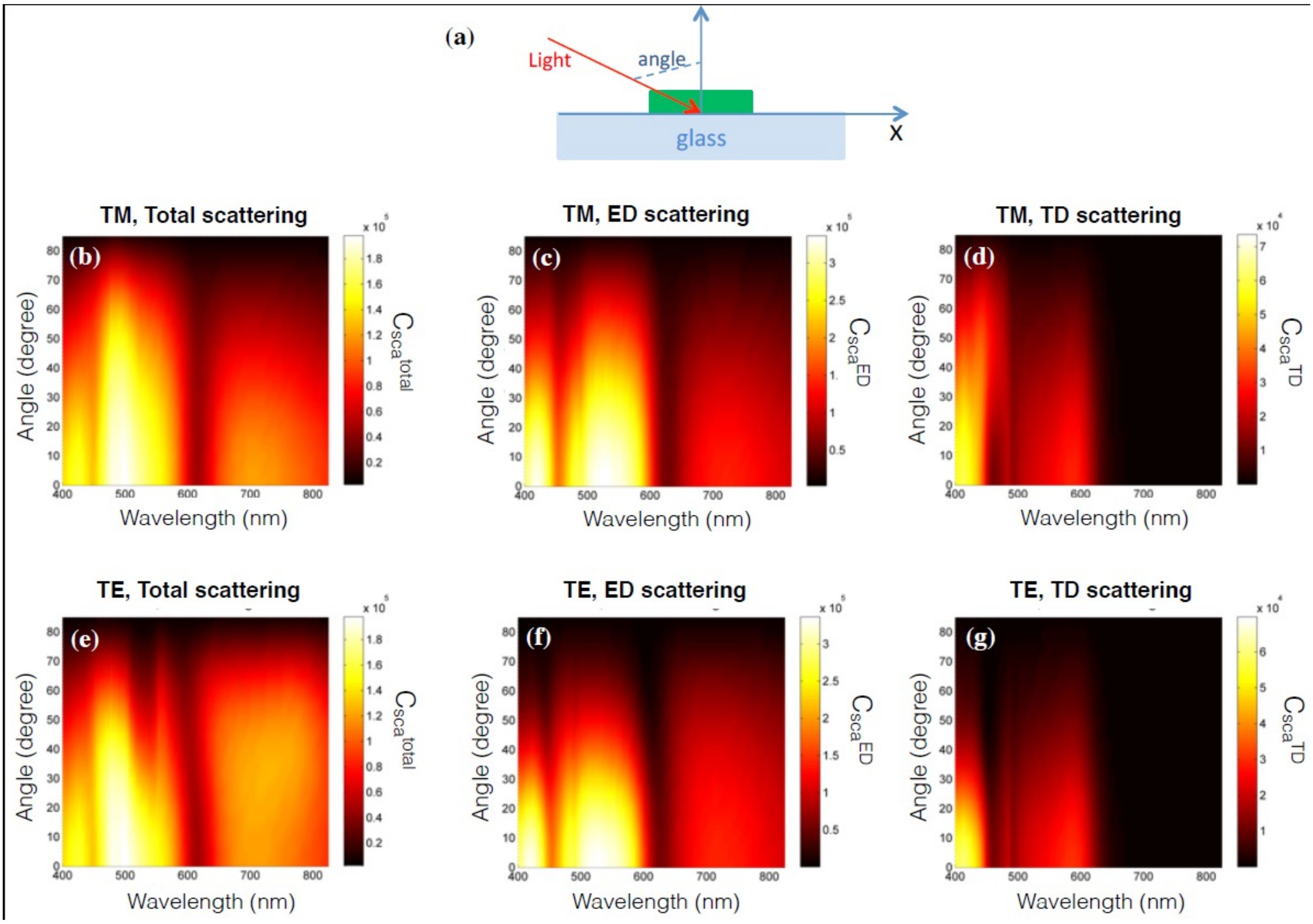

Supplementary Figure 2: **Numerical results for light scattering by silicon nanodisks at oblique incidence**. (a) Schematic of the simulation setup; (b, e) the total scattering cross-sections for TE and TM polarizations; (c,f) Cartesian electric and (d,g) toroidal dipoles contributions calculated by DDDA method for a silicon nanodisk with diameter 270 nm. These results demonstrate independence from incident angle and polarization.

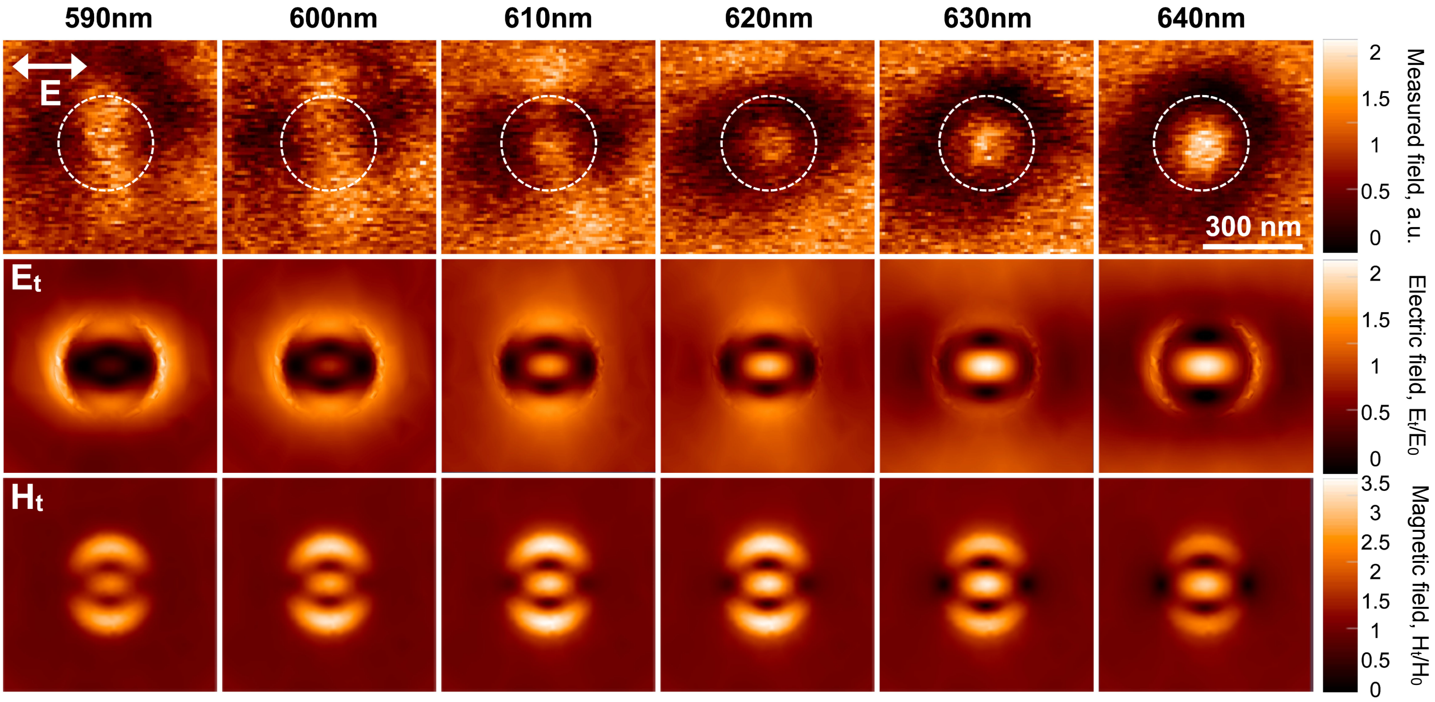

Supplementary Figure 3: **Near-field distribution around the silicon nanodisk with a height of 50 nm and diameter of 285 nm**. The top row shows experimental NSOM measurements while the middle and bottom rows show normalized calculated transversal electric and magnetic near-field respectively on top of the disk. White dashed lines in the experimental images indicate the disk position.

Supplementary Methods

Analysis of a spherical electric dipole response. For further insight into the origin of the scattering cancelations, we can consider a simplified situation of light scattering by a hypothetical spherical particle where we take scattering contributions only from the spherical electric dipole mode excited inside the sphere [see Fig.2]. The internal electric field of the spherical electric dipole can be analytically expressed as follows [1]

$$\mathbf{E}_{sph} \propto d_E(1,1) N_{e11}^{(1)} =$$
$$= d_E(1,1)\left(2\frac{j_1(\rho)}{\rho}\cos(\varphi) P_1^1(\cos\theta)\,\hat{\mathbf{e}}_r + \cos(\varphi)\frac{dP_1^1(\cos\theta)}{d\theta}\frac{1}{\rho}\frac{d}{d\rho}\left[\rho j_1(\rho)\right]\hat{\mathbf{e}}_\theta - \sin(\varphi)\frac{P_1^1(\cos\theta)}{\sin\theta}\frac{1}{\rho}\frac{d}{d\rho}\left[\rho j_1(\rho)\right]\hat{\mathbf{e}}_\varphi \right) \quad (1)$$

with

$$d_E(1,1) = \frac{n_{sph} j_1(\rho)\left[\rho h_1^{(1)}(\rho)\right]' - n_{Si} h_1^{(1)}(\rho)\left[\rho j_1(\rho)\right]'}{n_{sph}^2 j_1(n_{sph}\rho)\left[\rho h_1^{(1)}(\rho)\right]' - h_1^{(1)}(\rho)\left[n_{sph}\rho j_1(n_{sph}\rho)\right]'} \quad (2)$$

and $\rho = kR_{sph}$ being size parameter, n_{sph} is refractive index of the particle, $j_l(x)$ is spherical Bessel function, $P_1^1(\cos\theta)$ is associated Legendre polynomial [1].

Multipole expansion in vector spherical harmonic. Electromagnetic properties of silicon nanodisks were numerically studied by using CST Microwave Studio. In the Canonical basis we perform a multipole expansion of the scattered field of the nanodisk into vector spherical harmonics, which form a complete and orthogonal basis allowing the unique expansion of any vectorial field. To calculate electric $a_E(l,m)$ and magnetic $a_M(l,m)$ spherical multipole coefficients, we project the scattered electric field $\mathbf{E}_{sca}$ on a spherical surface, enclosing the silicon nanodisk centered at the symmetric point, onto vector spherical harmonics based on the following relations [2]:

$$a_E(l,m) = \frac{(-i)^{l+1}kR}{h_l^{(1)}(kR)E_0\sqrt{\pi(2l+1)(l+1)l}}\int_0^{2\pi}\int_0^{\pi} Y_{lm}^*(\theta,\phi)\hat{\mathbf{r}}\cdot\mathbf{E}_{sca}(\mathbf{r})\sin\theta\, d\theta\, d\phi$$
$$a_M(l,m) = \frac{(-i)^{l}kR}{h_l^{(1)}(kR)E_0\sqrt{\pi(2l+1)}}\int_0^{2\pi}\int_0^{\pi} \mathbf{X}_{lm}^*(\theta,\phi)\cdot\mathbf{E}_{sca}(\mathbf{r})\sin\theta\, d\theta\, d\phi \quad (3)$$

where R is the radius of the enclosing sphere, k is the wavevector, $h_l^{(1)}$ is the Hankel function with the asymptotic of the outgoing spherical wave, E_0 is the amplitude of the incident wave, Y_{lm} and $\mathbf{X}_{lm}$ are scalar and vector spherical harmonics. Due to azimuthal symmetry of the silicon nanodisk the amplitude of the scattering coefficients with opposite m indices are identical, $|a_{E,M}(l,m)| = |a_{E,M}(l,-m)|$ [1].

For the anapole excitation discussed in this paper, the dominant contribution to the scattering is given by the spherical electric dipole $C_{sca} \propto |a_E(1,1)|^2$.

Cartesian multipoles and discrete dipole approximation. Additional multipole analysis of scattering were performed using the decomposed discrete dipole approximation (DDDA) [3,4]. In this approach the scattering object is replaced by a cubic lattice of electric dipoles with the polarizability α_j. The total number of dipoles is N. The corresponding dipole moment $\boldsymbol{p}_j$ induced in each lattice point j (with the radius-vector $\boldsymbol{r}_j$) is found by solving the coupled-dipole equations. The Cartesian multipole moments of the scattering object (electric dipole moment $\mathbf{p}$, electric quadrupole moment $\hat{Q}(\boldsymbol{r}_0)$, magnetic dipole moment $\mathbf{m}(\boldsymbol{r}_0)$, toroidal dipole moment $\mathbf{T}(\boldsymbol{r}_0)$, and magnetic quadrupole moment $\widehat{M}(\boldsymbol{r}_0)$ located at a point $\boldsymbol{r}_0$) are simply calculated from the space distribution of $\boldsymbol{p}_j$

$$\mathbf{p} = \sum_{j=1}^{N}\boldsymbol{p}_j; \qquad \hat{Q}(\boldsymbol{r}_0) = \sum_{j=1}^{N}\hat{Q}^j(\boldsymbol{r}_0); \qquad \mathbf{m}(\boldsymbol{r}_0) = \sum_{j=1}^{N}\boldsymbol{m}_j(\boldsymbol{r}_0); \quad (4)$$

$$\widehat{M}(\boldsymbol{r}_0) = \sum_{j=1}^{N}M^j(\boldsymbol{r}_0); \qquad \mathbf{T}(\boldsymbol{r}_0) = \sum_{j=1}^{N}\boldsymbol{T}_j(\boldsymbol{r}_0); \quad (5)$$

where the corresponding Cartesian multipole moments associated with the single electric dipole $\boldsymbol{p}_j$, are under the summation symbols. Analytical expressions for these multipole moments are presented as

$$\hat{Q}^j(\boldsymbol{r}_0) = 3\left((\boldsymbol{r}_0 - \boldsymbol{r}_j) \otimes \boldsymbol{p}_j + \boldsymbol{p}_j \otimes (\boldsymbol{r}_0 - \boldsymbol{r}_j)\right), \qquad \boldsymbol{m}_j(\boldsymbol{r}_0) = -\frac{i\omega}{2}[(\boldsymbol{r}_0 - \boldsymbol{r}_j)\times \boldsymbol{p}_j], \tag{6}$$

$$\hat{M}^j(\boldsymbol{r}_0) = -\frac{i\omega}{3}\{[(\boldsymbol{r}_0 - \boldsymbol{r}_j)\times \boldsymbol{p}_j] \otimes (\boldsymbol{r}_0 - \boldsymbol{r}_j) + (\boldsymbol{r}_0 - \boldsymbol{r}_j) \otimes [(\boldsymbol{r}_0 - \boldsymbol{r}_j)\times \boldsymbol{p}_j]\}, \tag{7}$$

$$\boldsymbol{T}_j(\boldsymbol{r}_0) = -\frac{i\omega}{10c}\left[\left((\boldsymbol{r}_0 - \boldsymbol{r}_j)\cdot \boldsymbol{p}_j\right)(\boldsymbol{r}_0 - \boldsymbol{r}_j) - 2(\boldsymbol{r}_0 - \boldsymbol{r}_j)^2 \boldsymbol{p}_j\right], \tag{8}$$

where ω is the angular frequency of scattered light.

The scattered electric field in the far field zone is presented by [5]

$$\mathbf{E}_S(\boldsymbol{r}) = \frac{k_0^2 e^{ik_d(r-\mathbf{n}\mathbf{r}_0)}}{4\pi\varepsilon_0 r}\Big([\mathbf{n}\times[\mathbf{p}\times\mathbf{n}]] + \frac{ik_d}{6}\left[\mathbf{n}\times\left[\mathbf{n}\times\hat{Q}(\boldsymbol{r}_0)\mathbf{n}\right]\right] + \frac{1}{v_d}[\mathbf{m}(\boldsymbol{r}_0)\times\mathbf{n}] + \frac{ik_d}{2v_d}[\mathbf{n}\times\hat{M}(\boldsymbol{r}_0)\mathbf{n}] + \frac{ick_d}{v_d}[\mathbf{n}\times[\mathbf{T}(\boldsymbol{r}_0)\times\mathbf{n}]]\Big), \tag{9}$$

where k_0, c, and k_d, v_d are the wavenumber and light velocity in vacuum, and the wavenumber and light phase velocity in the medium surrounding the scatterer, respectively. ε_0 is the vacuum dielectric constant, $\mathbf{n}$ is the unit vector directed along the radius vector $\boldsymbol{r}$. Using the scattered electric field we calculated the total and angular distributions of scattered powers and to estimate contributions of every multipole moment [4]. Note that here $\hat{M}(\boldsymbol{r}_0)$ is symmetrical and traceless [5]. Contribution of the electric octupole moment in our case is significantly small, so we excluded it from the consideration.

Supplementary References:

1. Bohren and Huffman, *Absorption and Scattering of Light by Small Particles.* (Wiley, 2007).
2. Jackson, *Classical Electrodynamics* [Third Edition], (Wiley, 1998).
3. Evlyukhin, A.B., Reinhardt, C., and Chichkov, B.N., Multipole light scattering by nonspherical nanoparticles in the discrete dipole approximation. *Phys. Rev. B* **84**, 235429 (2011).
4. Evlyukhin, A.B., Reinhardt, C., Evlyukhin, E., and Chichkov, B.N., Multipole analysis of light scattering by arbitrary-shaped nanoparticles on a plane surface. *J. Opt. Soc. Am. B* **30** 2589 (2013).
5. Chen, J., Lin, J. Ng, Z., and Chan, C. T., Optical pulling force. *Nature Photonics* **5**, 531–534 (2011).
